# Proteomic Insights on the Metabolism of *Penicillium janczewskii* during the Biotransformation of the Plant Terpenoid Labdanolic Acid

**DOI:** 10.3389/fbioe.2017.00045

**Published:** 2017-07-31

**Authors:** Isabel Martins, Adélia Varela, Luís M. T. Frija, Mónica A. S. Estevão, Sébastien Planchon, Jenny Renaut, Carlos A. M. Afonso, Cristina Silva Pereira

**Affiliations:** ^1^Instituto de Tecnologia Química e Biológica António Xavier, Universidade Nova de Lisboa (ITQB NOVA), Oeiras, Portugal; ^2^Instituto Nacional Investigação Agrária e Veterinária, Oeiras, Portugal; ^3^Instituto de Investigação do Medicamento (iMed.ULisboa), Faculdade de Farmácia, Universidade de Lisboa, Lisboa, Portugal; ^4^Environmental Research and Innovation (ERIN) Department, Luxembourg Institute of Science and Technology, Belvaux, Luxembourg

**Keywords:** labdanolic acid, stress response, terpenoids biotransformation, proteomics, *Penicillium janczewskii*, cytochrome P450 monooxygenase

## Abstract

Plant terpenoids compose a natural source of chemodiversity of exceptional value. Many of these compounds own biological/pharmacological activity, others are regarded as unique chemical skeletons for the synthesis of derivatives with improved properties. Functional chemical modification of terpenoids through biotransformation frequently relies on the use of Ascomycota strains, but information on major cellular responses is still largely lacking. *Penicillium janczewskii* mediates a stereo-selective hydroxylation of labdanolic acid (LA)—terpenoid found abundantly in *Cistus ladanifer*—producing 3β-hydroxy-labdanolic acid with yields >90%. Herein, combined analyses of mycelial and extracellular differential proteomes demonstrated that the plant terpenoid increased stress responses, especially against oxidative stress (e.g., accumulation of superoxide dismutase) and apparently altered mitochondria functioning. One putative cytochrome P450 monooxygenase differentially accumulated in the secretome and the terpenoid bioconversion was inhibited *in vivo* in the presence of a P450 inhibitor. The stereo-selective hydroxylation of the plant terpenoid is likely mediated by P450 enzymes, yet its unequivocal identity remains unclear. To the best of our knowledge, this is the first time that proteomics was used to investigate how a plant terpenoid impacts the metabolism of a filamentous fungus during its efficiently biotransformation. Our findings may encourage the development of new strategies for the valorization of plant natural resources through biotechnology.

## Introduction

Terpenoids compose a dissimilar group of natural compounds (>25,000 compounds identified so far) that structurally are saturated and unsaturated cyclic and aliphatic hydrocarbons with variable degrees of oxygenation, including alcohols, aldehydes, ketones, and carboxylic acids (Tholl, 2015). Typically these molecules are distributed into subclasses according to the number of isoprene units in their structure. Terpenoids are often regarded as valuable natural resource with far-reaching applications, from flavors, fragrances, pigments, and agrochemicals to pharmaceutical drugs. In particular, among the subclass of the labdane-type diterpenes (20 carbon atoms) several show cytotoxicity (Yang et al., 2010), bactericidal (Singh et al., 2010; Ghosh and Rangan, 2014), antioxidant (Kapewangolo et al., 2015), antifungal (Hawas et al., 2015; Mendoza et al., 2015), and anti-inflammatory properties (Huang et al., 2015; Kapewangolo et al., 2015).

**Figure 1:**
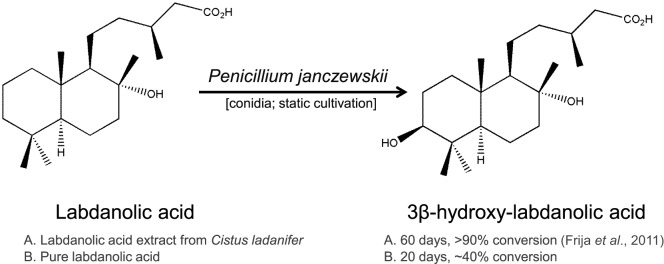
Hydroxylation of labdanolic acid at the 3β position mediated by *Penicillium janczewskii*.

Due to its richness in phytochemicals, the genus *Cistus* has been proposed as a model for the biosynthesis of labdane-type diterpenes and also as a natural source of high value pharmacological products (Papaefthimiou et al., 2014). In particular, labdanolic acid (LA) (Figure 1), a diterpene extracted in large quantities from the *Cistus ladanifer* L. (“Rock-rose”) (Martins et al., 2014a), displays anti-inflammatory properties (Jayaprakasam et al., 2007) and is used as a precursor compound in the chemical synthesis of numerous valuable compounds, e.g., Ambrox^®^ (the prototype compound of all ambergris odorants) (Bolster et al., 2001). This emphasizes well the importance to better understand how this subclass of plant terpenoids impacts on the metabolism of microbes that may be used for their modification. In opposition to the chemical modification of terpenoids, biotransformation renders products with a “natural” label and usually requires mild-reaction conditions avoiding also generation of toxic byproducts (Bicas et al., 2009). Therefore, biotransformation constitutes a reference method for producing new terpenoid derivatives with improved properties (Silva et al., 2013; Schrader and Bohlmann, 2015). The capacity of fungi, as well as bacteria, to transform terpenoids yielding stereo- and regio-selective products and/or compounds functionalized at particular positions has been well documented (e.g., Frija et al., 2011; Mutafova et al., 2016). In a previous study, we reported that three Ascomycota fungal strains (out of the eight tested) were able to transform LA (Frija et al., 2013). In particular, *Penicillium janczewskii* mediated a stereo-selective hydroxylation of LA yielding 3β-hydroxy-labdanolic acid as the major product (Figure 1). This terpenoid derivative would be, in general, difficult to attain by classical chemical methods and, so far, has never been identified in natural extracts (Frija et al., 2013). 3β-Hydroxy-labdanolic acid was used for the chemical synthesis of, e.g., 3β-hydroxy-labd-8(17)-en-15-oic acid—a moderate bactericidal compound, previously identified in the stems of *Moldenhawera nutans* (Frija et al., 2013).

Most studies on fungal biotransformation of terpenoids have essentially identified/characterized the terpenoid derivatives being produced, whereas their general impact on the fungal metabolism and the biotransformation pathways remain largely unknown, hampering the biotechnological development of this field. This study aims to specifically analyze *P. janczewskii* metabolism during the biotransformation of LA. With this goal in mind, herein we performed a differential proteomic analysis of both mycelial and extracellular proteomes, complemented by *in vivo* inhibition assays of cytochrome P450 monooxygenase activity. LA apparently increased stress responses, especially against oxidative stress in *P. janczewskii* cultures and its stereo-selective hydroxylation likely involves a P450 enzyme.

## Materials and Methods

### Materials and Chemicals

The LA extract was obtained from twigs of *Cistus ladanifer* as previously described (i.e., crude acidic fraction of which ≈16% is LA) (Frija et al., 2013). Chemicals were purchased from Sigma Aldrich (USA), except electrophoresis reagents such as dithiothreitol (DTT), Triton X-100, 3-[(3-cholamidopropyl) dimethylammonio]-1-propanesulfonate (CHAPS) and IPG buffers that were from GE Healthcare (Sweden). All solvents used were of the highest analytical grade and water was obtained from a Milli-Q system (Millipore).

### Fungal Strain and Cultivation Conditions

*Penicillium janczewskii* K. M. Zalessky (CBS#3498) conidia were harvested and maintained as frozen suspensions (Carvalho et al., 2009). Cultures were initiated with 10^5^ conidia/mL and incubated without agitation in the dark at 27°C (nine replicates). The fungal cultures (20 mL) were grown in a mineral minimal medium (Carvalho et al., 2009) supplemented, per liter, with 10 g of glucose, 0.25 g of urea, and 2.5 g of the LA extract (hereafter defined solely as LA medium), which was added from a concentrated stock in ethanol prior to the media sterilization. Final concentration of LA in this medium was *ca*. 0.04 mg/mL. Controls without LA were prepared in similar conditions. To keep the experimental conditions similar to those used in our previous study (Frija et al., 2013), after 60 days of incubation both mycelia and filtrate were recovered, immediately frozen in liquid nitrogen and stored at −80°C until further analyses (Martins et al., 2014b). Small aliquots of the culture broths were used to evaluate qualitatively by thin layer chromatography (TLC, see below) the biotransformation of the plant LA. The presence of 3β-hydroxy-labdanolic acid was evaluated by mass spectrometry (see below). In addition, to evaluate catalase activity in the secretome of cultures grown in LA medium, the ability of extracellular proteins (concentrated using 10 kDa centrifugal devices and resuspended in 0.1 M phosphate buffer, pH 7) to decompose H_2_O_2_ *in vitro* (Aebi, 1984) was qualitatively verified by the release of oxygen bubbles.

### *In Vivo* Inhibition of Cytochrome P450

Cultures were initiated from spores (10^5^/mL) in the minimal media containing, per liter, 10 g of glucose, 0.25 g of urea, and 0.02 g of pure LA (Sigma) (LA standard medium) (5 mL, 27°C, dark, no agitation). Metyrapone (2.0 mM) was added to half of these cultures due to its capacity to inhibit *in vivo* cytochrome P450 monooxygenase activity (Strauber et al., 2003). Culture filtrates were collected at the 21^st^ day of incubation, immediately extracted with diethyl ether and LA quantitatively analyzed by high performance liquid chromatography (HPLC) (see below). The identity of the compounds was validated by mass spectrometry (see below).

### Thin Layer Chromatography

Culture filtrates were extracted with diethyl ether (1:1; six times), the ensuing extracts dried under soft nitrogen flow, and re-solubilized in diethyl ether (1 mL). The extracts (10 µL) were resolved in TLC silica gel 60 F_254_ plates (Merck) with diethyl ether/*n*-hexane (3:1) as the mobile phase. At the end of the TLC run, the plates were air dried, immersed in a solution of 10% of phosphomolybdic acid in ethanol, and revealed by heating at 80–100°C during 5 min.

### High Performance Liquid Chromatography

Labdanolic acid was analyzed by HPLC, using an Alliance 2695 Waters chromatographer (Waters Corporation, Milford, MA, USA), connected to a Differential Refractometer (Bromma, Sweden) detector. Data acquisition was accomplished with the Empower 2 software (Waters). Chromatographic separation was undertaken using a Symmetry C18 column (4.6 mm × 250 mm), 5 µm particle size (Waters) set at 28°C. Elution was carried out isocratically with water/acetonitrile (10:90) at a flow rate of 1.0 mL/min, and the injection volume was 95 µL. The retention time of the LA was compared with that of a pure standard (Sigma) for identification, and the peak area was used for quantification. LA retention time was 5.7 min, and the established quantification limits were 0.125–1 mg/mL.

### High Performance Liquid Chromatography–Electrospray Ionization–High Resolution Mass Spectrometry (HPLC-ESI-HRMS)

The diethyl ether extracts of the fungal cultures grown in the standard LA medium were analyzed by microLC–MS using a Triple TOF 6600 MS system (Sciex) equipped with the DuoSprayTM ion source. Chromatographic separation was carried out in the Eksigent ekspert nanoLC425 in microflow using an HALO C18 (50 mm × 0.5 mm, 2.7 µm particle size, 90 Å) column from Eksigent at the flow rate of 10 µL/min. The mobile phase consisted of a solution of 0.1% formic acid (solvent A) and a solution of acetonitrile containing 0.1% formic acid (solvent B), set as follows: 20% B in 2 min, followed by a linear gradient of 20–95% B in 12 min, 2 min of 95% B, 2 min to return to the initial conditions, and 5 min to re-equilibrate the column. MS was operated in positive ionization mode, with TOF MS scan with an *m*/*z* range 100–1,000 for 500 ms for a total cycle time of 0.5 s. MS data were processed using the PeakView software using the extracted-ion chromatogram for the compounds of interest. For compound identity a Δ(*m*/*z*) ≤ 5 ppm as well as a low noise/signal ratio, was considered.

### Protein Extraction

Established methods were used to extract mycelial (Martins et al., 2013) and extracellular proteins (Martins et al., 2014b). Mycelial [100 mg powder mixed with 20 mg poly(vinyl)polypyrrolidone from Merck] and extracellular proteins (concentrated using 10 kDa centrifugal devices) were precipitated with cold acetone containing 10% w/v trichloroacetic acid and 60 mM DTT. The washed pellets were dissolved in similar buffers (7 M urea, 2 M thiourea, 4% w/v CHAPS, 60 mM DTT, and 1.0% v/v of matching IPG buffer) except that 1% (w/v) Triton X-100 was also added to the buffer used in the extracellular proteins, and finally clarified by centrifugation.

### Two-dimensional Gel Electrophoresis (2DE)

Protein extracts were quantified using bovine serum albumin (BioRad) as a standard. Mycelial and extracellular protein samples (80 and 50 µg, respectively) were loaded in precast 13 cm non-linear IPG strips (GE Healthcare) (pH 3–10 NL and 3–5.6 NL, respectively). Protein isoelectric focusing, SDS-PAGE, staining, image acquisition, and analysis were done as previously reported (Martins et al., 2013, 2014b). The gels were stained with flamingo dye (BioRad, USA) for image acquisition but for spot excision colloidal coomassie blue (Fluka, Switzerland) was used instead and higher quantities of protein were loaded, namely, 180 and 100 µg for mycelial and extracellular protein fractions, respectively. For each condition, three biological replicates (each accounting three batch cultures) were done.

### Data Acquisition and Image Analyses

Gels stained with the flamingo dye were scanned in a Fuji TLA-5100 scanner to generate 64 bit images. The images were analyzed in SameSpots v2.0 (non-linear dynamics) accordingly to manufacturer’s instructions and included gel alignment to a reference image. Spot detection was refined by manual edition whenever necessary, and protein spots with areas lower than 1,500 were excluded from the analysis. Differential protein spots in the mycelial proteome were identified using SameSpots software (ANOVA *p*-value < 0.05). Aiming at a stringent statistical analysis (Valledor et al., 2010), normalized spot volumes were used for calculating mean values, SD, and CV across the two secretome gel sets (fungal grown in the LA medium or the control medium) (Data Sheet S1 in Supplementary Material). Only those spots showing high consistency between replicate gels (CV below 15%) were used to generate the list of *p*-values for the individual spots (ANOVA, X-Stat). This allowed to pinpoint the spots that were differentially accumulated in the secretome (Data Sheet S1 in Supplementary Material).

### Protein Identification

Differential protein spots on LA in both sub-proteomes were manually excised from gels and processed using the Ettan Digester robot of the Ettan Spot Handling Workstation (GE Healthcare) (Carvalho et al., 2013). Samples (0.7 µL) were then spotted on MALDI-TOF target plates (Applied Biosystems), before the deposit of 0.7 µL CHCA (7 mg/mL in ACN 50%, TFA 0.1%). Peptide mass determinations were carried out using the Applied Biosystems 5800 Proteomics Analyzer (Applied Biosystems) using established protocols (Carvalho et al., 2013; Martins et al., 2013, 2014b). Proteins were identified, with ProteinPilot, by searching against the NCBInr database (restricted to fungi taxonomy, 1,291,260 sequences) with Mascot v2.3 (Matrix Science). Homology identification was retained with probability set at 95%. All identifications were confirmed manually.

### Protein Functional Classification

The identified proteins were classified into categories according to the Munich Information Centre for Protein Sequences Functional Catalog.^1^ For proteins with no assigned function, homology searches were performed using the BlastP program against all non-redundant protein sequences deposited in the NCBI database^2^ to attribute putative functions. The cellular location of the extracellular proteins was predicted using WoLF-PSORT (Horton et al., 2007).

## Results

**Figure 2:**
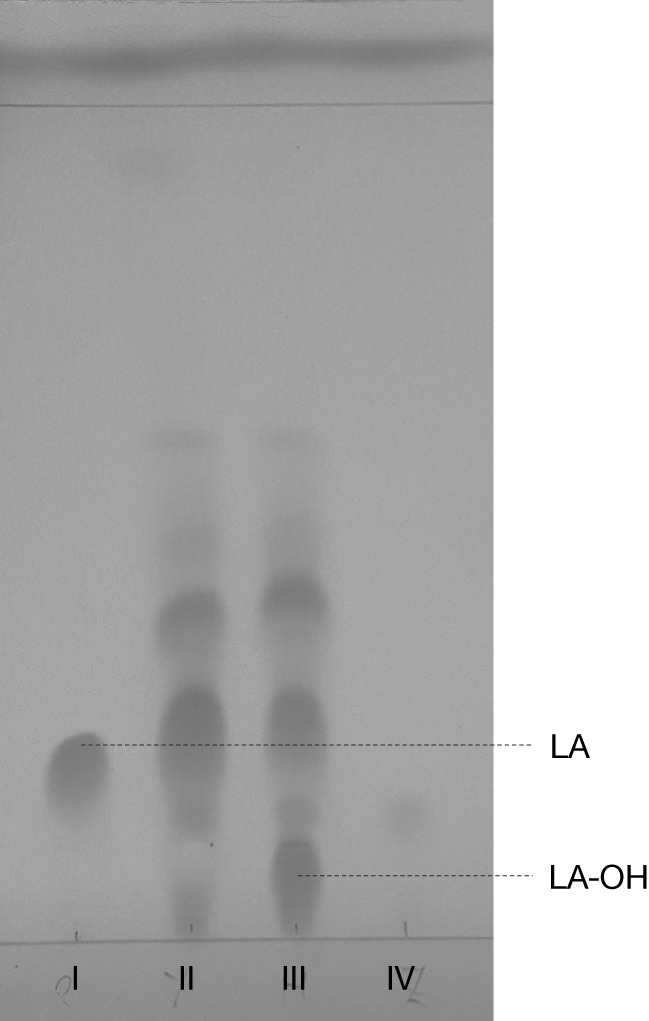
Thin layer chromatogram of *Penicillium janczewskii* cultures during the biotransformation of labdanolic acid (LA) to 3β-hydroxy-labdanolic acid (LA-OH): pure commercial LA (I); diethyl ether extract of *P. janczewskii* cultures in the LA medium at the incubation time (time 0) (II) and after 60 days of growth (III); and diethyl ether extract of the negative control (IV, i.e., *P. janczewskii* cultures after 60 days of cultivation in media without the LA extract). [Eluent: diethyl ether/*n*-hexane (3:1).]

Similar to our previous study (Frija et al., 2013), herein we observed that *P. janczewskii* mediates a stereo-selective hydroxylation of LA yielding predominantly 3β-hydroxy-labdanolic acid (Figure 2) that displays an ion mass (*m*/*z*) of 363.2506 [LA]Na^+^ (data not shown). In these static cultures (20 mL), not all the LA was hydroxylated after the two months of incubation (Figure 2, lane III).

**Figure 3:**
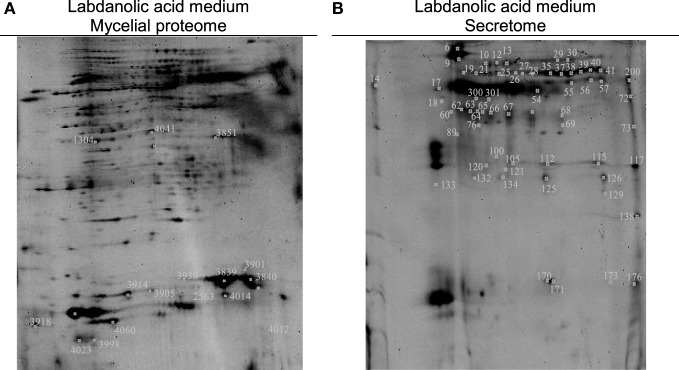
Two-dimensional gels obtained for the mycelia **(A)** and secretome **(B)** of *Penicillium janczewskii* cultures grown for 60 days in the presence of labdanolic acid. The spots highlighted showed a statistically significant increase (*p*-value < 0.05) during cultivation in media supplemented with the plant terpenoid (protein identifications are depicted in Tables 2 and 3).

In this study, *P. janczewskii* mycelial and extracellular sub-proteomes after growth in LA or control media were analyzed using a 2DE approach (Figure 3). In both sub-proteomes, the protein extraction yields were slightly higher in the LA medium compared to the control yet retrieving a comparable number of total protein spots, notwithstanding the long incubation time used (Table 1). Comparative proteomic analyses were undertaken to identify in each sub-proteome, the protein spots that increased during growth in the LA medium compared to the control medium (i.e., differential accumulation, ANOVA analysis, *p* < 0.05). The number of differentially accumulated proteins spots in the mycelial proteome and the secretome was respectively, 18 and 20, retrieving 17 and 7 unique protein species (Data Sheet S2 in Supplementary Material); herein depicted in Tables 2 and 3, where proteins are grouped by functional categories. Regardless that the genome of this fungus is still unsequenced, a large fraction of the differential protein spots in either sub-proteome matched protein species annotated in *Penicillium* species already sequenced (e.g., *P. chrysosporium, P. digitatum*, and *P. marnafreii*) (Data Sheet S2 in Supplementary Material). Most of the remaining protein identifications were matched against sequences from *Aspergillus* species. The proteomic analysis provided a snap-shot view of major alterations provoked by the supplementation of the growth media with LA compared to control conditions (i.e., absence of LA). In general, the LA extract increased stress responses in *P. janczewskii*, especially against oxidative stress [e.g., superoxide dismutase (SD) and NAD(P)H-quinone oxidoreductase, Table 2]. Catalases were also show to differentially accumulate (Table 3); consistent with the detection of catalase activity (i.e., hydrogen peroxide decomposition assay) in the extracellular protein extracts from cultures grown in LA medium. A detailed description of important cellular events likely associated with the identified differential protein species (Tables 2 and 3) is presented in the Section “Discussion.”

**Table 1:** Protein extraction yield and number of identified spots in the 2DE gels.

Media/sample		Protein yield	Number of spots	
			Total number	Differentially accumulated
Labdanolic acid	Mycelial	3.76 ± 2.06 µg/g FW	714 ± 19	18
	Secretome	2.99 ± 0.43 µg/mL	209 ± 18	20
Control	Mycelial	2.69 ± 0.42 µg/g FW	733 ± 23	–
	Secretome	1.44 ± 0.29 µg/mL	209 ± 6	–

*FW, fresh weight*.

**Table 2:** *Penicillium janczewskii* mycelial proteins identified in the 2DE differentially accumulated protein spots [fold change (FC)] in the labdanolic acid medium when compared to the control medium.

Spot #^a^	FC^b^	*p*-Value^c^	NCBI (gi)	Protein name	Organism	Subcellular location^d^
**Mitochondria**						
3851	4.2	0.002	238491872	Outer mitochondrial membrane protein porin	*Aspergillus flavus*	Mitochondria
4041	4.8	0.002	255954547	Succinate dehydrogenase	*Penicillium digitatum*	Mitochondria
**Defense and pathogenesis**						
3914	3.4	0.0007	144952798	16 kDa allergen	*P. chrysogenum*	Extracellular
3918	4.6	0.0008	425766829	Allergen Asp f 15	*P. digitatum*	Extracellular
3905	3.9	0.0007	51702151	Superoxide dismutase (SD) [Cu–Zn]	*A. nidulans*	Cytosol
3938	4.0	0.005	51702125	SD [Cu–Zn]	*A. flavus*	Cytosol
**Protein and nucleotide**						
4012	3.9	0.003	70984978	Nucleoside diphosphate kinase	*A. fumigatus*	Cytosol/nuclear
3901	3.3	0.0004	255942649	Ribosomal protein	*P. chrysogenum*	Cytosol
2563	3.6	0.009	93140599	Peptidyl-prolyl cis–trans isomerase	*Ustilago maydis*	Unknown
**Miscellaneous**						
1304	1.9	0.023	119497213	NADH-quinone oxidoreductase	*Neosartorya fischeri*	Cytosol
3998^e^	5.3	0.0008	255936587	Unknown function protein	*P. chrysogenum*	Cytosol
4014	6.7	0.0002	121719277	BYS1 domain protein	*A. clavatus*	Extracellular
4023	8.8	0.0002	238506637	Unknown function protein	*A. flavus*	Extracellular
3998^e^	5.3	0.0008	238506637	Unknown function protein	*A. flavus*	Extracellular
3840	2118	0.0003	255936199	Unknown function protein	*P. chrysogenum*	Extracellular
3839	317.8	0.0003	255936199	Unknown function protein	*P. chrysogenum*	Extracellular
4060	2.8	0.013	255936199	Unknown function protein	*P. chrysogenum*	Extracellular

*Protein species are grouped by their functional classification. One differential protein spot has not retrieved any protein identification*.

*^a^Numbered accordingly to the 2DE map of mycelia proteins of Penicillium janczewskii*.

*^b^FC calculated using SameSpots software*.

*^c^ANOVA p-value calculated using SameSpots software*.

*^d^Predicted by WoLF-PSORT (Horton et al., 2007)*.

*^e^Protein spots that retrieved more than one protein identification*.

**Table 3:** *Penicillium janczewskii* extracellular proteins identified in the 2DE differentially accumulated protein spots [fold change (FC)] in the labdanolic acid (LA) medium when compared to the control medium.

Spot #^a^	FC^b^	*p*-Value^c^	NCBI accession (gi)	Organism	Protein name	SigP^d^
**LA biotransformation**						
126	1.17	0.006468	346326084	*Cordyceps militaris*	Putative cytochrome P450 monooxygenase^f^	N
**Fungal cell wall remodeling**						
115	1.23	0.000704	70985687	*Aspergillus fumigatus*	GPI-anchored cell wall β-1,3-endoglucanase EglC	Y
117	1.2	0.037162				
**Protease/nitrogen**						
60	0.83	0.002876	169770151	*A. oryzae*	Leucine aminopeptidase 2	Y
105^e^	0.92	0.036227				
134	0.91	0.048728				
76^e^	0.81	0.034241	121698850	*A. fumigatus*	Secreted dipeptidyl peptidase	Y
129	1.22	0.004811				
**Defense**						
14	0.87	0.013724	82754305	*Penicillium citrinum*	Catalase	Y
41	1.16	0.042395				
73	1.12	0.007392				
76^e^	0.81	0.034241				
**Miscellaneous/unknown**						
300	1.43	0.001527	302505575	*Arthroderma benhamiae*	FAD/FMN-containing isoamyl alcohol oxidase Mrea-like	N
105^e^	0.92	0.036227	328854667	*Melampsora larici-populina*	Unknown function protein	N

*Protein species are grouped by their functional classification. Eight differential protein spots, out of 20, have not retrieved any protein identification*.

*^a^Numbered accordingly to the 2DE map of mycelia proteins of Penicillium janczewskii*.

*^b^FC calculated as the ratio between the normalized volumes of the spots in the LA- and control media*.

*^c^ANOVA p-value calculated using X-stat*.

*^d^Predicted by WoLF-PSORT (Horton et al., 2007)*.

*^e^Protein spots that retrieved more than one protein identification; Spot 105 also retrieved identification as unknown function protein*.

*^f^The protein shows significant homology to CYP503B1 from *Beauveria bassiana* [gi: 667649775; score: 313; expect: 3e^−100^; identities: 183/361 (51%); positives: 221/361; gaps: 7 3/361 (19%)]*.

**Figure 4:**
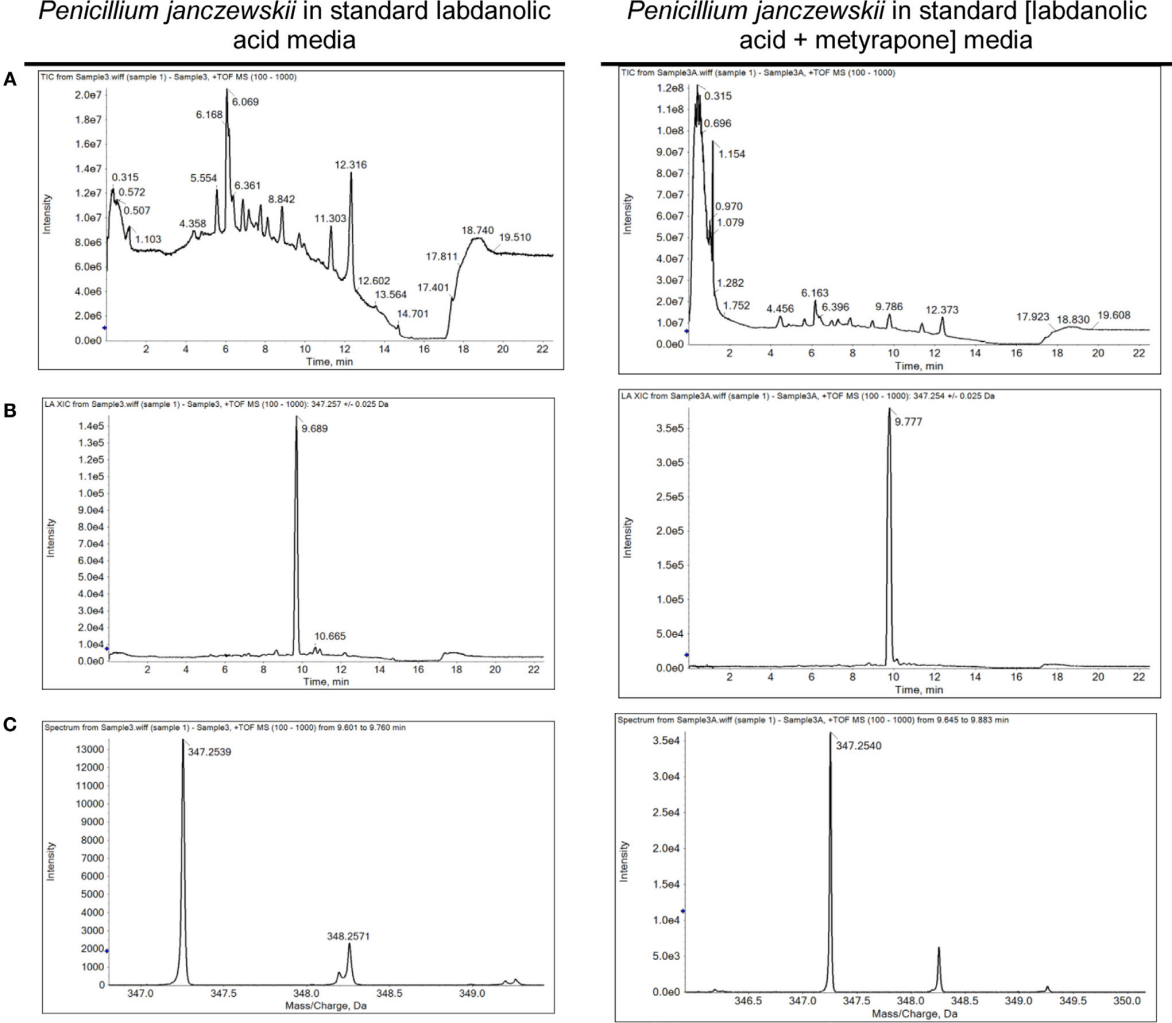

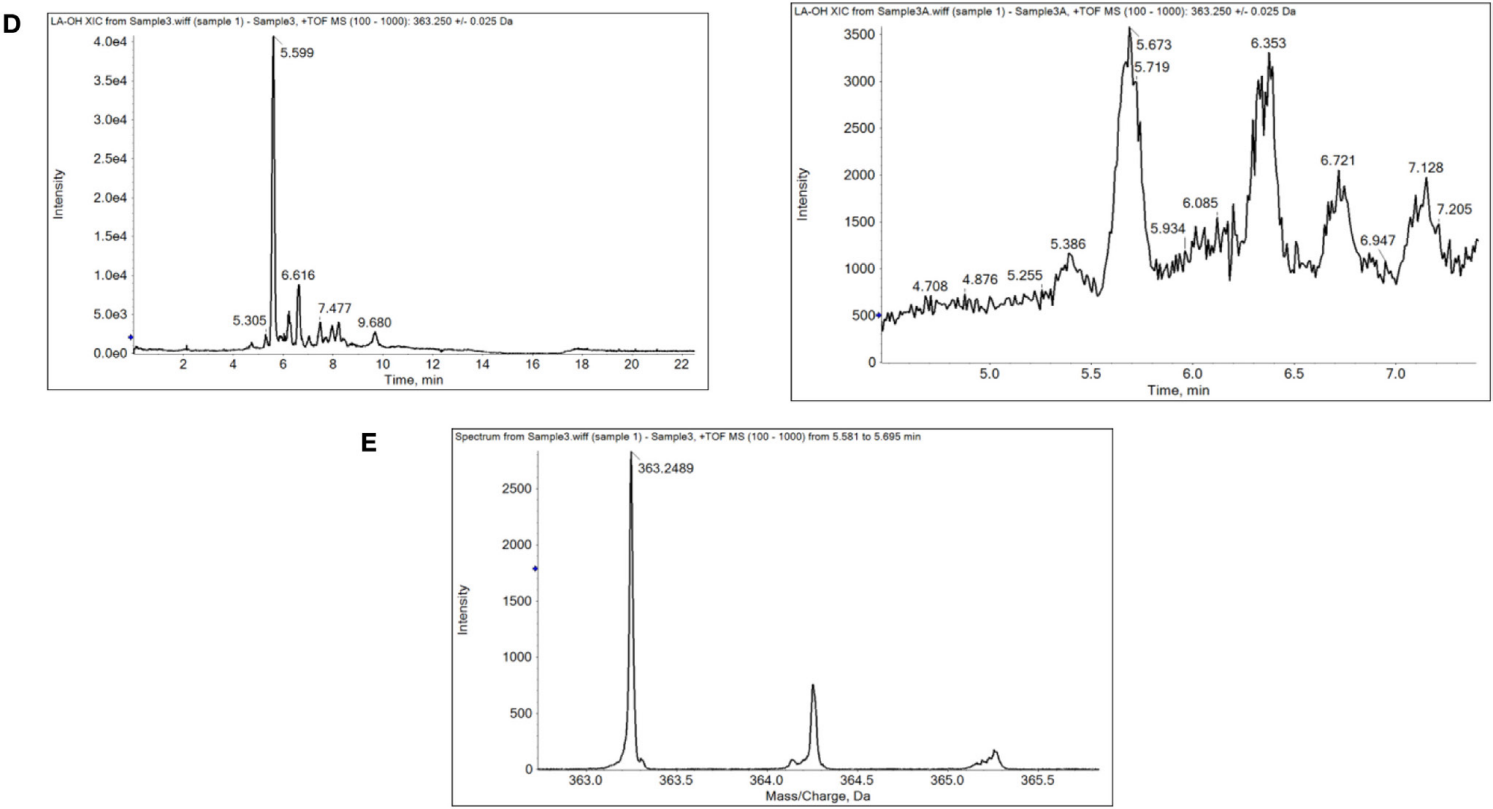
Spectrometric analyses of the biotransformation of pure labdanolic acid (LA) in the absence or presence of metyrapone (inhibitor of P450 activity) in *Penicillium janczewskii* cultures. Total ion chromatograms **(A)**, extracted-ion chromatogram (XIC) obtained for LA **(B)** and for 3β-hydroxy-labdanolic acid **(D)**; and the isotopic pattern obtained for LA **(C)** and for 3β-hydroxy-labdanolic acid **(E)**, illustrating the data collected during the HPLC-ESI-HRMS analyses.

Among the set of identified protein species up-accumulated in the presence of LA, only a putative cytochrome P450 monooxygenase (P450s or CYPs) [spot 126, fold change (FC) = 1.17, Table 3] came forward as capable of mediating the stereo-selective hydroxylation of the terpenoid. To investigate the involvement of P450 enzymes in the biotransformation of LA, their activity was inhibited *in vivo* using pure LA (0.02 mg/mL) as a substitute of the plant extract (containing ≈0.04 mg/mL of LA). In the absence of P450 inhibitors, the bioconversion rate of pure LA reached *ca*. 40% at the 21^st^ day of incubation, whereas in their presence the conversion was significantly inhibited, dropping to *ca*. 18% (Table 4, HPLC). To further verify that the stereo-selective hydroxylation of LA has occurred, the presence of 3β-hydroxy-labdanolic acid was verified by its ion mass using high resolution mass spectrometry. The MS results showed that the ion mass of LA could be detected in cultures grown in either the absence or presence of the P450 inhibitor (Figures 4B,C), whereas that of 3β-hydroxy-labdanolic acid could only be detected in cultures devoid of the P450 inhibitor (Figures 4D,E).

**Table 4:** Biotransformation yields of pure labdanolic acid (LA) in the absence or presence of metyrapone (inhibitor of P450 activity) in *Penicillium janczewskii* cultures as determined by liquid chromatography.

Cultures	LA% consumption	LA^a^ ion mass intensity	LA-OH^b^ ion mass intensity
LA standard media	40.19 ± 7.90	8.09e^4^	1.59e^4^
LA standard media + metyrapone	18.18 ± 6.65	2.56e^5^	^c^

*Labdanolic and 3β-hydroxy-labdanolic acid ion masses detected in the cultures by spectrometry considering only compounds reporting an error lower than 5 ppm and a low noise/signal ratio*.

*^a^LA detected *m*/*z* = 347.257 [M + Na]^+^, C_20_H_36_O_3_Na*.

*^b^3β-Hydroxy-labdanolic acid (LA-OH) detected *m*/*z* = 363.2506 [M + Na]^+^, C_20_H_36_O_4_Na*.

*^c^Below the detection limits*.

## Discussion

### LA Stereo-Specific Hydroxylation by *P. janczewskii*

Terpenoids chemical modifications through biotransformation, in particular stereo-selective hydroxylation of non-activated carbons in their structures, provide derivatives which would be usually difficult to achieve by chemical methods (Frija et al., 2013; Schrader and Bohlmann, 2015; Kemper et al., 2017). Fungi can biotransform terpenoids in particular product(s) and at specific rate(s) through multiple reactions strongly influenced by the cultivation conditions (Frija et al., 2011; Ghasemi et al., 2014; Mutafova et al., 2016). However, production yields of each distinct product from a stereo-selective hydroxylation of the terpenoid are usually very low (2–20%). Herein *P. janczewskii* could efficiently hydroxylate LA; regardless that not all LA was converted to LA-OH (Figure 2, lane III). When pure LA (0.02 mg/mL) was used as a substitute of the plant extract (containing ≈0.04 mg/mL of LA), the bioconversion rate reached *ca*. 40% at the 21^st^ day of incubation (Table 4; Figure 4).

In most studies reported so far, the terpenoid containing medium is inoculated with mycelia (previously grown in a standard growth medium) and agitated batch or fed-batch cultivation conditions are used (Frija et al., 2011; Schrader and Bohlmann, 2015; Mutafova et al., 2016). Terpenoids are known to play major roles in plant defense mechanisms (Gershenzon and Dudareva, 2007; Mutafova et al., 2016), probably explaining why the LA plant extract inhibited the germination of *P. janczewskii* conidia in the agitated cultures. In our previous study, even at static conditions, germination of conidia was only observed in three out of the eight fungal strains tested (Frija et al., 2011). Several studies support the catabolic potential of *P. janczewskii*, e.g., for producing secondary metabolites (Madi and Katan, 1998), exo-inulinases (Pessoni et al., 2007), xylanases (Terrasan et al., 2010, 2016, 2017), and galactosidases (Zhang et al., 2011), as well as its ecological relevance in modulating plant–fungi interactions, either antagonizing (Madi and Katan, 1998) or stimulating (Kwasna, 2004) important plant pathogenic fungi.

Comparative proteomic analyses (Figure 3) of *P. janczewskii* sub-proteomes allowed the identification of 24 unique protein species that differentially accumulated during growth on the LA medium (Tables 2 and 3; Data Sheet S2 in Supplementary Material). A small number of distinct proteins spots (Tables 2 and 3) retrieved identical functional annotations that might result from posttranslational modifications of the same gene product (i.e., proteolysis, glycosylation, and phosphorylation) (Mann and Jensen, 2003) or from the presence of sequence-related isoforms encoded by distinct paralogs (Vödisch et al., 2009). Since *P. janczewskii* fully annotated genome sequence is not yet available on the basis of MS data alone, one cannot discriminate between these two possibilities.

### Stress Responses to LA Exposure in *P. janczewskii* Cultures

The biological effect of labdane-type terpenoids (e.g., manoyl oxides) is usually associated with their lipophilicity, and the presence of specific end groups that may interact with cellular membranes altering their permeability and ultimately leading to severe damage and lipid peroxidation (Matsingou and Demetzos, 2007). Terpenoids usually lead to accumulation of intracellular reactive oxygen species (ROS) (Liu et al., 2013) and increase oxidative stress response both at transcript (e.g., *Grosmannia clavigera* expose to pine extract) (Hesse-Orce et al., 2010) and protein levels (e.g., *Saccharomyces cerevisiae* incubation with d-limonene) (Liu et al., 2013). ROS can severely damage the cells, causing oxidative damage of membranes, DNA and proteins, hence their levels are tightly regulated in fungi through a set of antioxidant responses both enzymatic (e.g., SOD, glutathione peroxidase, and catalase) and non-enzymatic (e.g., ubiquitinol and glutathione) (Aguirre et al., 2006; Marschall and Tudzynski, 2016). Several proteins species associated with antioxidant response, namely, SOD and catalase showed increased levels in the LA medium compared to control conditions (Tables 2 and 3). In particular, SODs (spots 3905 and 3938, FC = 3.9 and 4.0, respectively) that catalyze the dismutation of the superoxide radical, producing oxygen and/or hydrogen peroxide, constitute the first line of defense against oxidative stress in fungi (Belozerskaya and Gessler, 2007). The produced hydrogen peroxide can be subsequently decomposed to water and molecular oxygen by catalases. Two catalases showed a minor increase in *P. janczewskii* secretome on LA (spots 41 and 73, Table 3). This finding is consistent with their frequent detection in fungal secretomes undergoing oxidative stress conditions (Adav et al., 2012; Martins et al., 2014b). Supplementation of the growth media with catalase has been shown to reduce the ROS level and alleviated cell growth inhibition during *S. cerevisiae* cultivation in media containing d-limonene (Liu et al., 2013).

Growth media supplementation with sclareol lead to increased oxygen consumption on *Botrytis cinerea* cultures, probably due to altered mitochondrial function (Mendoza et al., 2015). In addition, α-pinene was shown to affect strongly the energy metabolism of mitochondria isolated from maize (Abrahim et al., 2003; Mendoza et al., 2015). This terpenoid provoked the uncoupling of oxidative phosphorylation and the inhibition of electron transfer, likely due to unspecific damage in the inner mitochondrial membrane (Abrahim et al., 2003; Mendoza et al., 2015). Similar findings were reported for β-pinene which strongly inhibited respiration in yeast cells and in their isolated mitochondria, probably due to its capacity to increase membrane fluidity (Uribe et al., 1985). Herein, two mitochondrial proteins species increased in *P. janczewskii* mycelial proteome on LA, namely, outer mitochondrial membrane protein porin (spot 3851, FC = 4.2) and succinate dehydrogenase (spot 4041, FC = 4.8) (Table 2). The last enzyme catalyzes the oxidation of succinate to fumarate with the reduction of ubiquinone to ubiquinol, coupling the citric acid cycle and the electron transport chain in the inner mitochondrial membrane. Mitochondrial ubiquinol acts as antioxidant molecule, participating in non-enzymatic anti-ROS mechanisms (Bai et al., 2003; Dinkova-Kostova and Talalay, 2010). LA apparently altered fungal mitochondrial proteins and led to the generation of ROS, notwithstanding its effects were less severe than those induced by more lipophilic terpenoids such as α-pinene and limonene (Abrahim et al., 2000).

NAD(P)H-quinone oxidoreductase is a widely distributed FAD-dependent flavoprotein that promotes obligatory reductions of quinones (as well as of other aromatic molecules) thus dropping their intracellular levels and minimizing the generation of reactive oxygen intermediates by redox cycling (Dinkova-Kostova and Talalay, 2010). It plays major antioxidant roles fighting oxidative stress and is induced as a stress-responsive protein, e.g., in human cells exposed to *Angelica sinensis* extracts (Dietz et al., 2008) and in *A. nidulans* (AN0297) expose to either menadione (Pusztahelyi et al., 2011) or ionic liquids (Martins et al., 2013). LA also increased this stress response protein in the mycelia of *P. janczewskii* (spot 1304, FC = 1.9, Table 2).

A link between oxidative stress and increase of virulence has been suggested (Nikolaou et al., 2009), which might explain the differential accumulation of allergens in the mycelial proteome on LA, namely, the 16 kDa allergen and the allergen Asp f 15 (FC = 3.4 and FC = 4.6, respectively) (Table 2). Other stress-related proteins that increased on LA include the ubiquitous and highly conserved nucleoside diphosphate kinase (spot 4012, FC = 3.9, Table 2). This enzyme is crucial for the cellular homeostasis of nucleosides triphosphate and diphosphate (Lee et al., 2006). As an example, the transcription of its encoding gene is developmentally regulated and induced during stress response in *Aspergillus* spp. (Malavazi et al., 2006).

In this study, major accumulation of the unknown function protein containing a Bys1 domain (spot 4014, FC = 6.7, Table 2) was found in the mycelial proteome on LA. The antifungal caspofungin has been also shown to increase the levels of a protein containing a Bys 1 domain in *A. fumigatus* (Cagas et al., 2011). This class of proteins has been associated to morphogenesis, in particular the encoding gene increases during the yeast phase of the dimorphic fungus *Blastomyces dermatitidis* (Burg and Smith, 1994), notwithstanding the protein could not be identified in a proteomic deep analysis of dimorphism in *P. marneffei* (Chandler et al., 2008).

The differential protein spot number 3998 (FC = 5.3, Table 2) in the mycelial proteome retrieved two protein identifications, hampering the differential analysis. Both were identified as unknown function protein: one as hypothetical protein with no characterized function; and, the other with high homology to Grg1—a general stress protein involved in lifespan control, which was also identified in the spot 4023 (FC = 8.8). *grg1* has been previously identified in dormant conidia of *A. fumigatus* (Sugui et al., 2008) and levels increased during starvation and asexual development in *Neurospora crassa* (*ccg-1*) (Xie et al., 2004). In this study, at the end of the cultivation conidia were visible in the mycelial mat at the liquid air interface (data not shown).

Additional spots found to significantly increase in the mycelial proteome on LA media (3840, 3839, and 4060 with FC = 2,118, 317.8, and 2.8, respectively, Table 2), were however identified as uncharacterized proteins. Future functional and kinetic studies on fungal proteins are essential to elucidate their roles during fungal growth in media supplemented with the plant terpenoid.

### LA Hydroxylation

The differential accumulation of a putative P450 (spot 126, FC = 1.17) was found in the fungal secretome on LA (Table 3). This poorly characterized protein shows homology to a predicted P450 in *Cordyceps militaris* that is thought to contain only 290 amino acid residues. CYPs putative sequence lengths in *A. nidulans* (CYPome) were manually corrected from (286–750) to (417–607) amino acid residues (e.g., correction of incorrect exons and start/stop sequences) (Kelly et al., 2009). To the best of our knowledge, a similar analysis is missing in the CYPome of *C. militaris*, hence this predicted P450 possibly contains more than 290 residues. It shows also significant homology to CYP503B1 from *Beauveria bassiana* (Table 3); a class usually associated with the production of secondary metabolites in *A. nidulans* (Kelly et al., 2009; Moktali et al., 2012).

Genome sequencing projects on fungi are continuously revealing an increasing number of P450s (Park et al., 2008; Urlacher and Girhard, 2012; Chen et al., 2014), regardless the function of many remain unknown (Urlacher and Eiben, 2006; Chen et al., 2014). In fungi these enzymes play critical roles in the adaptation to specific ecological and/or nutritional niches by modifying potentially harmful chemicals, as well as in the production of an array of secondary metabolites. Interestingly, pine terpenoids increased the expression levels of several P450 genes of the secondary metabolism in *G. clavigera*, linking the encoded enzymes to their biotransformation (Lah et al., 2013).

P450s catalyze many reactions, such as oxygenation, dealkylation, epoxidation, reduction, dehalogenation and carbon hydroxylation (Urlacher and Girhard, 2012), including high stereo- and regio- selective hydroxylation of non-activated carbons, e.g., in *Cochliobolus lunatus* (Lah et al., 2011). They participate in the detoxification of a broad range of xenobiotics, particularly by mediating their initial modification (phase I), before transferase activity (conjugation with, e.g., glucoside, glucuronide, and sulfate—phase II) and cellular excretion (phase III) (Schäfer et al., 2004; Harms et al., 2011). None of the *P. janczewskii* differential accumulated proteins species on LA was identified as a transferase. However, conjugates of either LA or 3β-hydroxy-labdanolic acid, if any, might have been de-conjugated upon their cellular excretion through a reaction mediated by a catalase (Campoy et al., 2009). In fact, catalases were identified in multiple protein spots in the differential secretome (Table 3), and extracellular catalase activity was also detected.

The putative P450 identified herein (Table 3) is devoid of typical signal peptide sequence, notwithstanding its cellular compartmentalization is largely unknown (WoLF-PSORT predicted cellular locations: 12 plasma membrane bound; 6 extracellular; 3 endoplasmatic reticulum; 2 peroxisome; and 2 vacuolar). Though usually P450s are regarded as membrane-associated enzymes, they are increasingly being associated to the secretome (Druzhinina et al., 2012) as a consequence of autolysis or mycelial fragmentation (Kubicek, 2013) as well as secretion, e.g., secretome of *Postia placenta* growth on wood (5 days) (Vanden Wymelenberg et al., 2010). P450s found in fungal secretomes match multiple classes including some typically regarded as intracellular, emphasizing that current knowledge is still scarce for predicting the function of such uncharacterized enzymes. Several P450s have been linked to lignin degradation upon their detection in secretomes of fungi grown in lignocellulosic substrates (similar or twofold incubation time compared to that used here), e.g., *Phanerochaete carnosa* (Mahajan and Master, 2010) and *Trametes trogii* (Ji et al., 2012). In these studies, none of the P450 typical partners (i.e., NADPH-cytochrome P450 oxidoreductase) could be found in the fungal secretomes, similar to that observed here (Table 3).

The stereo-selective hydroxylation of pure LA was substantially inhibited when P450 activity was inhibited *in vivo*. LA hydroxylation levels were *ca*. 40 and 18% in the absence and presence of metyrapone (Table 4). The identity of 3β-hydroxy-labdanolic acid as well as LA were verified by mass spectrometry retrieving ion masses for [M + Na]^+^ of 363.2506 and 347.2570, respectively (full spectra and the isotopic pattern obtained for the extracts of the cultures is depicted in Figure 4). In the presence of metyrapone, the intensity of the ion chromatogram of 3β-hydroxy-labdanolic acid (Figure 4D) is comparable to the noise intensity; accordingly the corresponding isotopic pattern could not be extracted. This observation is consistent with the involvement of P450s in the biotransformation of LA.

Overall, the proteomic data provided novel information on the major cellular responses stimulated by the plant terpenoid during fungal growth, opening new perspectives of their role during plant–fungi interactions. Advanced processes for the biotransformation of terpenoids may require the use of antioxidants. We hope our study may promote the development of novel valorization strategies through biotechnology for the major terpenoids of the widespread resource *Cistus ladanifer* L.

## Author Contributions

IM executed all the proteome analyses and prepared the first draft of the manuscript. IM and AV executed the fungal cultivation and prepared the extracts for analytics. LF and ME produced the labdanolic extracts and provided analytical support. JR and SP performed the MS analyses for protein identification. CSP and CA conceived the study and performed critical revision of the data. CSP elaborated the final manuscript. All the authors contributed to the analysis and interpretation of data and have read and approved the final version of the manuscript.

## Conflict of Interest Statement

The authors declare that the research was conducted in the absence of any commercial or financial relationships that could be construed as a potential conflict of interest.

## Footnotes

^1^http://mips.helmholtz-muenchen.de/funcatDB/.

^2^http://blast.ncbi.nlm.nih.gov/Blast.cgi.
